# Nursing‐Led Oral Health Literacy Intervention and Changes in Dental Anxiety and Oral Health Behaviours Among Geriatric Patients With Dental Diseases: A Non‐Randomized One‐Group Pretest–Posttest Study

**DOI:** 10.1002/nop2.70972

**Published:** 2026-10-26

**Authors:** Abeer Ramadan Awade Gamaa, Mohamed Hussein Ramadan Atta, Mohamed Gamal Elsehrawy, Nesreen AbdelMonaem AbdelSataar AbouZeid, Naglaa Alsaied Mostafa Alfayomy

**Affiliations:** ^1^ Gerontological Nursing Department Faculty of Nursing, Damanhour University Damanhour City Egypt; ^2^ Psychiatric and Mental Health Nursing Department, Faculty of Nursing Alexandria University Alexandria City Egypt; ^3^ Nursing Department, College of Applied Medical Sciences Prince Sattam Bin Abdulaziz University Wadi Addawasir Saudi Arabia; ^4^ Nursing Administration and Education Department, College of Nursing Prince Sattam Bin Abdulaziz University Kharj City Kingdom of Saudi Arabia; ^5^ Nursing Administration Department, Faculty of Nursing Port‐Said University Port‐Said City Egypt; ^6^ Department of Medical and Surgical Nursing College of Nursing, Princess Nourah Bint Abdulrahman University Riyadh Saudi Arabia

**Keywords:** dental anxiety, gerontological nursing, health behaviour, older adults, oral health, oral health literacy, patient education

## Abstract

**Aim:**

To assess within‐group changes in dental anxiety, oral health knowledge and oral health behaviours following an oral health literacy intervention among older adults with dental diseases.

**Design:**

A non‐randomized one‐group pretest–posttest quasi‐experimental design.

**Methods:**

A convenience sample of 50 older adults participated in seven educational sessions addressing oral health, oral hygiene, nutrition, regular dental attendance and strategies for managing dental anxiety. Data were collected using the Saint Louis University Mental Status Examination, a sociodemographic questionnaire, the Oral Health Knowledge Scale, the Short Dental Anxiety Inventory and an oral healthcare behaviours questionnaire.

**Results:**

Following the intervention, participants had lower dental anxiety scores and higher oral health knowledge and oral health behaviour scores compared with baseline. Higher oral health knowledge was also associated with more favourable oral health behaviours.

**Conclusion:**

Lower dental anxiety and higher oral health knowledge and oral health behaviour scores were observed following the oral health literacy intervention among older adults with dental diseases. Because the study used a non‐randomized single‐group design, the findings should be interpreted cautiously.

**Implications for the Profession and/or Patient Care:**

Gerontological nurses can integrate oral health education and simple dental‐anxiety management strategies into routine care to support preventive oral health practices and engagement with dental services.

**Impact:**

Dental anxiety and limited oral health knowledge can hinder preventive dental care in later life. Following the intervention, participants had lower dental anxiety scores and higher oral health knowledge and oral health behaviour scores compared with baseline. The findings may inform nursing care for older adults in outpatient, primary care, community and long‐term care settings.

**Reporting Method:**

The study was reported in accordance with the Transparent Reporting of Evaluations with Nonrandomized Designs statement.

**No Patient or Public Contribution:**

Participants contributed study data but were not involved in the design, analysis, interpretation or manuscript preparation.

## 1 Introduction

The older adult population is projected to more than double from 8.4 million in 2020 to 22 million by 2050 (United Nations Population Fund [UNFPA] 2021). As the older adult population grows rapidly, older adults become more vulnerable to dental diseases. The risk of dental disease is increasing because they have age‐related physiological changes along with multiple chronic conditions, such as uncontrolled diabetes mellitus, which increases the risk of periodontitis by 86%. In contrast, other diseases, such as rheumatoid arthritis, are associated with poor oral care practices (Chan et al. 2021). Moreover, the use of multiple medications for chronic illness increases the risk of xerostomia as an adverse effect on oral health. Xerostomia is also associated with other oral problems, such as dental caries, gum disease and oral infections (Thomson et al. 2021).

Oral health problems among older adults are a significant public health issue worldwide. Older adults commonly experience oral health problems such as tooth loss, dental decay, gum inflammation (gingivitis), periodontal disease, dry mouth (xerostomia), oral sores and other dental issues (Shokry et al. 2018). The Global Oral Health Status Report (2022) estimates that about 3.5 billion people worldwide suffer from oral diseases, with untreated tooth decay being the most common condition (World Health Organization [WHO] 2022). However, older adults frequently overlook and neglect these problems (Janto et al. 2022). Consequently, oral health problems negatively affect the older adult's nutritional intake and general health, resulting in reduced chewing ability, weight loss, limited food choices and oral pain (Badewy et al. 2021). A decline in oral function has been connected to a deterioration in general physical abilities and frailty among older adults, potentially leading to disability and loss of independence (Gamaa et al. 2024).

Dental diseases also affect older adults' emotional and social well‐being, as they have implications for appearance, self‐confidence and the ability to communicate and interact with others, leading to social isolation and reduced social well‐being. Furthermore, oral health problems increase dental and medical costs, and it is necessary to increase older adults' literacy regarding the factors and behaviours that promote lifelong dental health (Mariño et al. 2020; Khamrin et al. 2021).

Dental anxiety (DA) is a significant barrier to oral health, especially among older adults (Appukuttan 2016). It involves adverse psychological reactions to dental visits, often stemming from previous painful or traumatic experiences. A study by Hassan et al. (2022) reported a high prevalence of DA among older adults (90.5%), with many being highly anxious or phobic. Dental anxiety among older adults is multifaceted and stems not only from general nervousness about daily oral hygiene, but also from specific memories and anxieties tied to dental practices and clinical settings. For example, adults often report that painful procedures—especially oral surgery and drilling—along with early negative experiences in dental care, continue to influence attendance behaviour even decades later (Pavičić et al. 2024). Additionally, anxiety triggered by the clinical environment—such as the sight of needles and drills, unfamiliar dental smells and perceived dentist behaviour—contrasts with the more routine stress or discomfort associated with daily brushing and flossing (Márton and Madléna 2024). This anxiety often leads to dental avoidance, resulting in poor oral hygiene, delayed care and reduced oral health‐related quality of life (Winkler et al. 2023).

Although dental anxiety may contribute to delayed or avoided dental care, older adults experiencing dental anxiety may still present to dental clinics when they experience pain, worsening oral symptoms or require professional assessment or treatment. Therefore, dental clinics may represent an important point of contact for identifying older adults experiencing dental anxiety and addressing their oral health needs through targeted interventions (Crego et al. 2014; Saanum et al. 2026). Nurses are well‐positioned to identify signs of anxiety through routine assessments and patient interactions. They can implement evidence‐based interventions such as relaxation techniques, patient education and psychological support to reduce anxiety before and during dental procedures. Moreover, nurses act as advocates by facilitating communication between patients and health care professionals, ensuring individualized care that addresses both physical and emotional needs. Recent studies emphasize the value of integrating nursing care into oral health promotion for older adults, noting that trained nursing staff can significantly increase dental service uptake and reduce anxiety‐related avoidance behaviours (Lee et al. 2022; Al‐Harbi and El Tantawi 2023).

Adopting effective oral health behaviours is crucial to achieving good oral health. Research has consistently demonstrated that oral health behaviours and knowledge about dental health are influenced by several factors, including educational level, socioeconomic status (SES) and family income (de Lucena et al. 2021; Çirakoğlu and Gökcek 2021). These behaviours include oral care practices, healthy food choices and the use of dental health services to prevent dental diseases (Marashi et al. 2023). Studies show that poor oral healthcare behaviours among older adults, such as improper brushing and infrequent tooth cleaning, are linked to oral health diseases (Khamrin et al. 2021; Marashi et al. 2023).

Oral health literacy (OHL) is essential for promoting oral health and improving quality of life. It refers to the degree to which individuals can obtain, process and understand basic health information and services needed to make appropriate oral health decisions (Chowdary et al. 2015). Higher OHL levels are associated with better oral health behaviours, status and self‐efficacy among older adults (Yu et al. 2024; Bashirian et al. 2023). Conversely, low OHL contributes to poor oral hygiene, limited use of preventive services and ineffective communication between older adult patients and dentists, thereby increasing dental anxiety and overall dissatisfaction with care (Silva‐Junior et al. 2021). Oral health literacy programs aim to enhance awareness, promote proper oral hygiene practices and prevent dental problems by encouraging daily brushing and flossing and regular dentist visits (Almabadi et al. 2021).

As a result, the World Health Organization and the FDI World Dental Federation emphasize the importance of maintaining oral health and function to support healthy aging among older adults (WHO 2015). A nurse‐led approach is pivotal in the successful implementation of oral health literacy interventions, particularly among older adults. Nurses, due to their continuous and close interactions with patients, are uniquely positioned to assess oral health needs, deliver tailored education and reinforce positive oral hygiene behaviours. Their leadership ensures that interventions are patient‐centered, culturally sensitive and aligned with individual health statuses. Moreover, nurses can coordinate multidisciplinary efforts, bridging gaps between dental professionals and other healthcare providers, to ensure comprehensive care. Their involvement can support the delivery of oral health programs, foster trust and encourage adherence to recommended oral health practices among older adults, potentially contributing to better oral health outcomes and overall well‐being (Daly and Smith 2015).

Despite increasing recognition of the importance of oral health literacy (OHL) in supporting oral health behaviours and better oral health outcomes among older adults, evidence regarding the effectiveness of nurse‐led oral health literacy interventions remains limited., particularly in addressing dental anxiety and oral health self‐management behaviours concurrently among older adults with dental diseases (Yu et al. 2024; Bøtchiær et al. 2024). Previous research has largely examined dental anxiety and oral health literacy as separate issues, with comparatively limited attention to integrated nursing approaches addressing both the psychological and behavioural dimensions of oral health. Moreover, evidence regarding such interventions among older adults with existing dental diseases remains limited (Hang and Chi 2025). This gap highlights the need for further research on evidence‐based, nurse‐led approaches to dental anxiety management and oral health self‐management among this vulnerable population.

Accordingly, the present study aimed to assess within‐group changes in dental anxiety, oral health knowledge and oral health behaviours following an oral health literacy intervention among older adults with dental diseases using a non‐randomized one‐group pretest–posttest design. Specifically, this study tested the following hypotheses:

*Participants will show a statistically significant increase in oral health knowledge post‐intervention compared to baseline (pre‐intervention)*.

*Participants will demonstrate a statistically significant reduction in dental anxiety levels post‐intervention compared to baseline*.

*Participants will exhibit statistically significant improvements in oral health self‐management behaviours post‐intervention compared to baseline*.

*Post‐intervention oral health knowledge will be positively correlated with oral health self‐management behaviours*.

## 2 Method

### 2.1 Research Design and Setting

This quasi‐experimental study employed a non‐randomized one‐group pretest–posttest design and was conducted at the Dental Clinic of the Damanhour National Medical Institute in Al‐Beheira Governorate, Egypt. The clinic is a well‐established healthcare facility serving both urban and rural communities, offering a wide range of dental services, including routine checkups, restorative procedures and specialized treatments. Staffed by qualified dental professionals and equipped with modern technology, the clinic provides a realistic, community‐based setting for studying oral health literacy, dental anxiety and self‐care behaviours among older adults.

### 2.2 Sampling and Participants

A convenience sample of 50 community‐dwelling older adults aged 60 years and above was recruited from the dental clinic. Participants were eligible if they were cognitively intact and capable of providing informed consent. Participants included those attending the dental clinic for the first time or those with limited previous utilization of oral health services, primarily due to dental‐related anxiety. Recruitment was conducted among older adults with dental diseases who presented to the dental clinic during the study recruitment period and met the predefined eligibility criteria. Although dental anxiety may discourage some older adults from seeking dental care, severe dental pain or an urgent need for treatment may sometimes override this fear and motivate individuals to attend the dental clinic despite their anxiety. Thus, the sample represented older adults with dental diseases who were accessible through the clinical setting. However, older adults with severe dental anxiety who completely avoided dental services and therefore did not present to the clinic could not be captured through this sampling approach. This potential selection bias was acknowledged as a limitation when interpreting and generalizing the study findings.

Individuals with cognitive impairment or severe mental health conditions that could interfere with participation or the ability to provide informed consent were excluded. Cognitive status was assessed through review of available health records and administration of the Arabic version of the Saint Louis University Mental Status (SLUMS) Examination. The SLUMS Examination, developed by Tariq et al. (2006) and is a 30‐point cognitive screening instrument that assesses multiple cognitive domains, including orientation, memory, attention, executive function, calculation, verbal fluency and visuospatial abilities. The Arabic version of the SLUMS was validated among Egyptian older adults by Abdelrahman and El Gaafary (2014), who reported acceptable validity and reliability, with a reliability coefficient of 0.723.

SLUMS scores were interpreted according to the established education‐adjusted scoring criteria. For participants with a high school education or higher, scores of 27–30 were considered within the normal cognitive range, scores of 21–26 indicated mild neurocognitive disorder, and scores of 1–20 indicated possible dementia. For participants with less than a high school education, including those who were unable to read or write, scores of 25–30 were considered within the normal cognitive range, scores of 20–24 indicated mild neurocognitive disorder, and scores of 1–19 indicated possible dementia. No separate researcher‐developed scoring modification was applied for illiterate participants. Because educational level and literacy may influence performance on cognitive screening instruments, the use of the established education‐adjusted SLUMS criteria was intended to minimize education‐related misclassification. SLUMS findings were used solely for cognitive screening and eligibility determination rather than as a definitive clinical diagnosis of neurocognitive disorder. Only participants whose scores fell within the normal range according to their educational category and who were capable of providing informed consent were included in the study (Table 1).

**TABLE 1:** SLUMS evaluation criteria.

Scoring	High school education	Less than high school education
Normal	27–30	25–30
Mild neurocognitive disorder	21–26	20–24
Dementia	1–20	1–19

A non‐randomized, one‐group pretest‐posttest design was used, in which all participants received the oral health literacy intervention. Outcomes were assessed at baseline (pretest) and 1 month after the intervention (posttest). The required sample size was calculated using G*Power software (Version 3.1.9.7) for a paired‐samples *t*‐test. Assuming a medium effect size (Cohen's *d* = 0.50), a two‐sided significance level of *α* = 0.05 and a statistical power of 80% (1−*β* = 0.80), the minimum required sample size was 34 participants.

To compensate for an anticipated attrition rate of approximately 30% during the intervention and 1‐month follow‐up period, the sample size was increased to 50 participants. This adjustment was based on the formula N_adjusted = *N*/(1 − *d*), where *N* is the minimum required sample size and *d* is the anticipated attrition proportion, yielding approximately 49 participants, which was rounded up to 50. The anticipated attrition was related to possible non‐attendance, loss to follow‐up or incomplete post‐test data. This approach was intended to maintain adequate statistical power to detect changes in dental anxiety, oral health knowledge and oral health self‐management behaviours (Julious 2009).

### 2.3 Tools

#### 2.3.1 Demographic and Clinical Data of Older Adults

The researchers developed this tool based on a review of relevant literature (Yu et al. 2024; Bashirian et al. 2023; Çirakoğlu and Gökcek 2021). It included three parts:

Part (1): demographic data were collected to provide a detailed understanding of the study participants and explore potential relationships between these factors and the study outcomes (40, 41). The data included age, recorded in years, to assess its impact on dental anxiety and oral health behaviours. Gender was categorized as male or female to examine differences between groups.

Part (2): Clinical data of older adults, such as medical history and self‐teeth assessment, including information regarding participants' chronic health conditions, was collected at baseline, including hypertension, diabetes mellitus, cardiovascular, liver, musculoskeletal, endocrine, respiratory and urinary conditions.

Part (3): Lifestyle such as dietary habits, oral hygiene practices (e.g., tooth brushing frequency, rinsing habits) and healthcare‐seeking behaviours (e.g., dental visits).

#### 2.3.2 Oral Health Knowledge Scale (OHK)

It was originally developed by Ahamed et al. (2015) to assess individuals' understanding of fundamental oral health concepts and practices. The scale comprises 10 multiple‐choice questions that assess knowledge of oral hygiene and the importance of maintaining oral health. Participants were asked to choose the correct answer for each item. Each correct response was scored as 1, while incorrect or ‘do not know’ responses were scored as 0. The total score reflects the participant's level of oral health knowledge, with higher scores indicating a better understanding of essential oral health practices.

The OHK has demonstrated validity and reliability in previous studies (Ahamed et al. 2015), and its consistent performance in measuring oral health knowledge in this context further supports its use in our research.

#### 2.3.3 The Short Dental Anxiety Inventory (S‐DAI)

It is a psychometric tool developed by Aartman (1998) to assess dental anxiety in patients; it consists of nine questions. The responses in the SDAI were collected using a 5‐point Likert scale (totally untrue, hardly true = 2, partly true = 3, quite true = 4, completely true = 5). The total score ranges from 9 to 45 points. The S‐DAI has demonstrated good psychometric properties, including strong internal consistency, with a Cronbach's alpha coefficient of 0.88. Evidence from previous research has also supported its validity for assessing dental anxiety.

#### 2.3.4 The Oral Healthcare Behaviours Questionnaire (OHB)

It was developed to assess the oral health‐related behaviours of older adults, drawing on insights from the relevant literature (An, Chen, et al. 2022). The questionnaire comprises nine items that cover critical aspects of oral hygiene and health practices, including dental cleaning, tooth brushing, use of dental floss, frequency of oral check‐ups, smoking habits, consumption of healthy foods, frequency of toothpaste use, use of fluoride toothpaste and use of toothpicks.

Participants were asked to reflect on their activities over the previous week and select one response for each item. The response options were structured across three levels: ‘Ever’ (2 points) indicated consistent engagement in the behaviour; ‘Sometimes’ (1 point) indicated occasional participation; and ‘Never’ (0 points) indicated no involvement in the activity. The total score for the OHB scale ranged from 0 to 18, with higher scores reflecting better oral health behaviours. This scoring system enabled a nuanced assessment of oral healthcare behaviours, facilitating the identification of patterns and gaps in practices among older adults. For statistical analysis, OHB scores were calculated using the mean and standard deviation.

### 2.4 Study Procedure

#### 2.4.1 Preparation Phase

The instruments were translated into Arabic using a forward‐ and back‐translation procedure to ensure linguistic equivalence between the original and Arabic versions. The initial translation was independently reviewed and back‐translated into English, and the original and back‐translated versions were compared to identify and resolve discrepancies and ensure conceptual and linguistic equivalence.

The Arabic versions were subsequently reviewed by a multidisciplinary panel of five experts, comprising two experts in Dentistry, one expert in Psychology, one expert in Education and one expert in Gerontological Nursing. The experts assessed the instruments for relevance, clarity, comprehensiveness and appropriateness for older adults with dental diseases. Their feedback was considered in refining the Arabic versions before their administration. Face validity was subsequently assessed through preliminary administration of the translated instruments to a small group of older adults from the target population to ensure that the items were clear, understandable and relevant to their experiences. Any observations regarding item clarity or comprehensibility were considered before the final administration of the instruments.

To evaluate the reliability of the instruments in the present study population, internal consistency was assessed using Cronbach's alpha. The coefficients were 0.88 for the OHB, 0.79 for the OHK and 0.83 for the S‐DAI, indicating acceptable to good internal consistency and supporting the reliability of the instruments in the current study.

Although standardized instruments for assessing oral health literacy (OHL), such as the REALD, TOFHLiD and HeLD, are available, their direct use was not considered feasible in the current study. These instruments may require relatively high levels of reading fluency, comprehension of dental terminology and familiarity with written assessment formats, which may present challenges for some older adults, particularly those with limited education or visual or cognitive difficulties. Furthermore, at the time of the study, suitable validated Arabic versions of these instruments were not available for the target population. Therefore, the Oral Health Knowledge Scale (OHK) and Oral Healthcare Behaviours Questionnaire (OHB) were selected to assess key measurable dimensions related to oral health literacy, specifically participants' knowledge and application of oral health information in their daily practices. We acknowledge that these instruments do not constitute a direct measure of overall oral health literacy.

Following the content validation process, the research team developed a structured oral health literacy program based on a comprehensive review of evidence‐based guidelines and relevant literature (27, 31, 32, 36). The same multidisciplinary panel of five experts, comprising two experts in Dentistry, one expert in Psychology, one expert in Education and one expert in Gerontological Nursing, reviewed the program to assess its relevance, clarity, comprehensiveness and appropriateness for the target population. The experts' recommendations were considered and incorporated into the final version of the program. The program was developed with the following objectives:

1. Educate older adult participants about the importance of oral health.
2. Teach practical skills for maintaining good oral hygiene.
3. Address common oral health issues in older adults.
4. Promote regular dental check‐ups.

#### 2.4.2 Interactive Activities

- Demonstrations of proper brushing and flossing techniques.
- Hands‐on practice with oral hygiene tools.
- Question‐and‐answer sessions to address participant concerns.

#### 2.4.3 Educational Materials

- Brochures and handouts with oral care tips.
- Illustrated guides showing step‐by‐step oral hygiene routines.
- Videos demonstrating proper techniques.

After the program was developed, revisions were made based on feedback from dental experts and professionals with experience in oral care. Subsequently, a pilot study was conducted with three patients to assess the feasibility and clarity of the intervention materials and procedures. The pilot study was covered by the same institutional ethical approval dated 21 December 2023. The three pilot participants were excluded from the final analytic sample.

#### 2.4.4 Oral Health Literacy (OHL) Intervention Implementation

The oral health literacy program was delivered over seven structured sessions, conducted twice weekly at the Dental Clinic of Damanhour National Medical Institute. Each session lasted approximately 45–60 min, combining interactive discussions, visual aids, demonstrations and hands‐on practice to ensure participant engagement and understanding. Each session began with a warm welcome to the participants, acknowledging their presence and creating a comfortable atmosphere. A summary of the previous session was provided to reinforce learning and ensure continuity. Efforts were made to ensure that the environment was conducive to learning, with participants comfortably seated and all necessary materials prepared in advance. The session also began with a clear outline of its objectives, helping participants understand what they would learn and what to expect.

#### 2.4.5 Training and Standardization of Interventions

The program was delivered by a team of six trained nurses who underwent a standardized 2‐week training workshop conducted by the researchers before the intervention. The training was designed to ensure comprehensive preparation and consistency in the delivery of the oral health educational intervention. It included a scientific review of the program content, including common oral health problems among older adults, daily oral hygiene practices and non‐pharmacological approaches to managing dental anxiety. The training also emphasized effective communication strategies tailored to older adults, instructional methods for conducting health education sessions, and hands‐on practice through simulation exercises.

The training was structured around the seven intervention sessions and covered the specific objectives, key content areas, teaching procedures, educational materials, interactive activities, demonstrations and practical applications for each session. The nurses were trained to deliver the same standardized content and to perform their assigned roles consistently across all sessions.

At the end of the training, the nurses' understanding of the intervention content and their ability to deliver the educational sessions were assessed through discussion, demonstration and simulation‐based practice. Feedback and clarification were provided by the researchers to ensure that all nurses achieved a consistent level of understanding and proficiency before participating in the intervention. The same intervention protocol, Arabic educational booklet, teaching materials, session sequence and standardized instructions were provided to all nurses.

The nurses' roles included:

- Demonstrating oral hygiene techniques, including tooth brushing, flossing and denture care.
- Facilitating interactive components, including question‐and‐answer discussions and practical applications.
- Distributing printed educational materials, including brochures and illustrated guides.
- Providing individualized support and follow‐up reminders during post‐session interactions.
- Encouraging open discussion to help older adults address dental anxiety and develop oral health self‐management skills.

Throughout the intervention, the researchers monitored adherence to the standardized protocol to promote consistency and fidelity in intervention delivery. Any questions or uncertainties regarding the intervention content or delivery procedures were discussed with the research team to maintain standardization across the interventionists.

Each session was conducted in small group settings (4–6 participants) to promote interaction and allow individualized attention. The intervention was held in a quiet, well‐lit meeting room within the dental clinic and all materials (e.g., anatomical models, hygiene kits, educational videos) were prepared in advance. The sessions were conducted exclusively by the nursing team; however, the entire implementation process was carried out under the close supervision of the research team. This supervisory role ensured adherence to the intervention protocol, maintained consistency and fidelity in session delivery, and supported the nurses in addressing participants' questions and challenges as needed.

A summary of session content (topics only) is provided in Table 2, with detailed content included in the Appendix to avoid redundancy and streamline the main text.

**TABLE 2:** Content outline of the oral health literacy program.

Session	Topic	Key content areas	Nurses' role in delivery
1	Introduction to Oral Health	Importance of oral health in overall well‐being Impact on physical, mental and social health Natural oral changes with aging	Led discussions on the role of oral health in general wellness Used visual aids to explain age‐related oral changes Assessed participants' baseline knowledge
2	Common Oral Health Issues in Older Adults	Gum disease: causes, symptoms, prevention Tooth decay and cavities Dry mouth (Xerostomia) Oral cancer awareness	Explained signs/symptoms and management strategies using patient‐friendly language Guided participants in identifying early warning signs Provided handouts for home reference
3	Dental Anxiety Among Older Adults	Definition and causes of dental anxiety Nonpharmacological strategies (e.g., relaxation, exposure)	Conducted group discussions on fears and misconceptions Demonstrated anxiety‐reduction techniques Created a supportive environment to build trust
4	Daily Oral Hygiene Practices	Brushing and flossing techniques Denture and appliance care Oral self‐examination	Demonstrated correct techniques using models Supervised hands‐on practice with tools Answered hygiene‐related questions in real‐time
5	Nutrition and Oral Health	The role of diet on oral health Foods to include/avoid Importance of hydration	Explained dietary guidelines for oral health Used real food examples to illustrate concepts Offered tips for managing dry mouth through nutrition
6	Regular Dental Visits	Value of routine dental check‐ups What to expect during a dental visit Addressing barriers (cost, transport)	Simulated dental visit steps to reduce fear Provided resources for affordable dental services Helped participants plan upcoming visits
7	Oral Health and Systemic Conditions	Oral‐systemic links (e.g., diabetes, heart disease, arthritis) Medications and Their Association with Oral Health When to communicate with their healthcare providers regarding their chronic conditions, medications and oral health concerns.	Explained how chronic conditions impact oral health Reviewed medication side effects Encouraged communication with healthcare providers

#### 2.4.6 Evaluation Phase

Data collection was carried out by members of the research team who were not involved in delivering the oral health literacy sessions. This separation of roles was intended to reduce potential measurement bias. Recruitment commenced on 4 March 2024, and the intervention sessions commenced on 16 March 2024 while recruitment was still ongoing. Recruitment was completed on 20 April 2024.

Pre‐intervention data—including demographic characteristics, oral health knowledge, oral healthcare behaviours and dental anxiety—were collected face‐to‐face during the participants' initial clinic visit. One month after completion of the program, post‐intervention data were collected to assess knowledge retention and changes in dental anxiety and oral health behaviours. The final 1‐month post‐intervention follow‐up assessment was completed on 15 June 2024. This approach was designed to assess changes in the study outcomes following the nursing‐led oral health literacy intervention (Figure 1).

**FIGURE 1:**
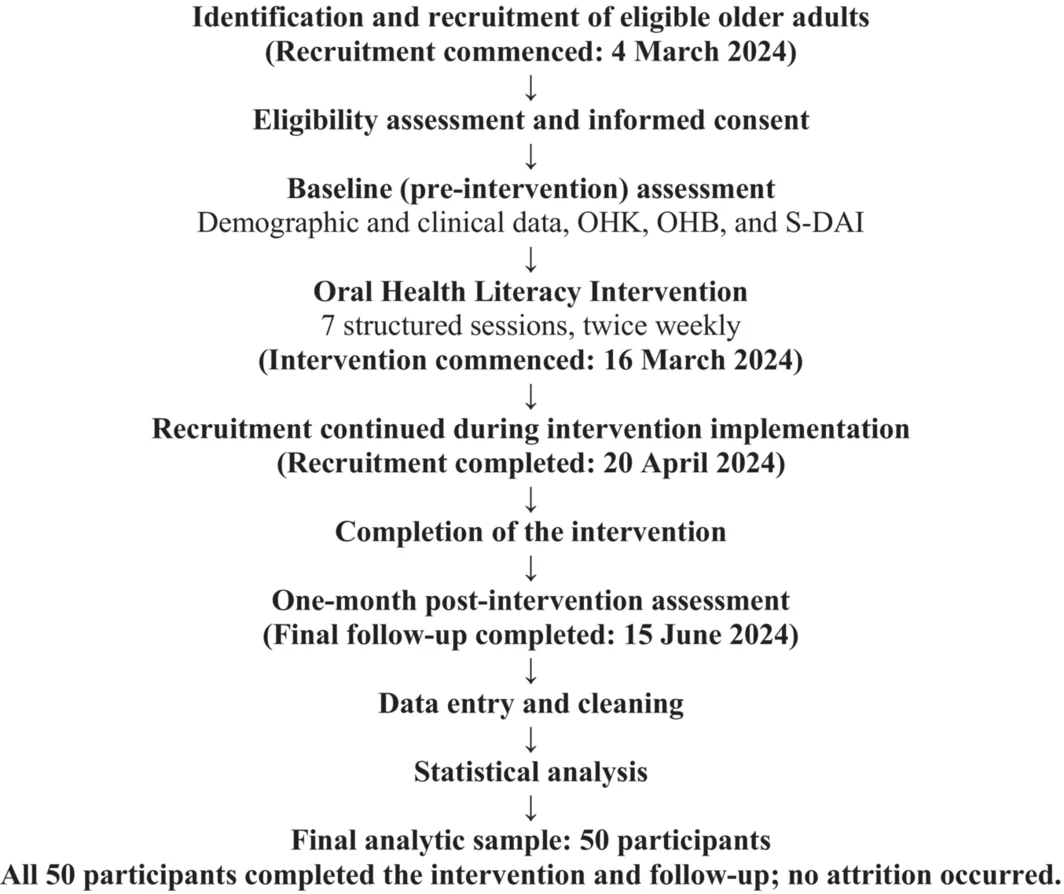
Study procedure flowchart.

#### 2.4.7 Quality Control Procedures

Several quality control procedures were implemented during data collection and data management to minimize potential errors associated with self‐reported data. Before data collection, the research team was trained on the standardized administration procedures of all study instruments and on how to provide neutral explanations without influencing participants' responses. The questionnaires were administered in a quiet and private setting, and participants were encouraged to respond independently based on their own experiences.

For older adults who required assistance because of reading difficulties, the items were read aloud using standardized wording, without explaining or suggesting a particular response. The researchers clarified the meaning of items only when necessary and avoided leading questions. Each completed questionnaire was reviewed immediately after completion for completeness, consistency and legibility, and any missing responses were identified and addressed when possible while the participant was still available.

During data organization, the completed questionnaires were coded using identification numbers rather than participants' names. The data were entered into a computerized database using standardized coding procedures and were checked for completeness, range errors and inconsistencies before statistical analysis. The research team reviewed the entered data against the original questionnaires to minimize data‐entry errors. These procedures were implemented to enhance the accuracy, completeness and consistency of the collected self‐reported data.

### 2.5 Ethical Framework

Before conducting the study, the study protocol was submitted to the Research Ethics Committee of the Faculty of Nursing, Damanhour University, Egypt for review and approval. Ethical approval was obtained on 21 December 2023 (approval No. 92‐a). Written informed consent was obtained from all participants after they were informed about the study objectives, procedures and methodology. Participants were informed that their participation was entirely voluntary and that they could withdraw from the study at any time without any consequences. Their data were collected anonymously and used solely for research purposes. The study was conducted in accordance with the ethical principles of the Declaration of Helsinki, as revised by the World Medical Association (WMA) in Fortaleza, Brazil, in 2013.

### 2.6 Statistical Analysis

Data were analyzed using IBM SPSS Statistics for Windows, Version 23.0 (IBM Corporate; Armonk, NY, USA). Frequencies and percentages were used to summarize categorical demographic characteristics, whilst continuous variables (dental anxiety, oral health knowledge and oral health self‐management behaviour scores) were reported as mean ± SDs. Statistical analyses were conducted systematically to test the study hypotheses and examine within‐group changes in the study variables between baseline and post‐intervention assessments. Paired‐samples *t*‐tests were then conducted within the single group to assess baseline‐to‐post‐intervention changes in dental anxiety (H1), oral health knowledge (H2) and oral health self‐management behaviours (H3). Normality of the change scores were evaluated formally (based on difference variables defined as post‐intervention minus baseline) before executing the paired‐samples *t*‐tests. There was no evidence of deviation from normality which tested through Shapiro–Wilk tests (*W* = 0.965, *p* = 0.148) within the paired difference scores used in these tests for dental anxiety so parametric paired testing is justified. In order to estimate the effect size associated with these pre–post changes beyond simple statistical significance, Cohen‐*d* effect sizes were computed for each paired outcome and categorized accordingly (0.2 = small, 0.5 = medium, 0.8 = large). The calculated mean differences were accompanied with 95% confidence intervals (95% CI) to estimate the range of precision as a function of the effect that the intervention would have on each outcome variable.

Independent‐samples *t*‐tests and one‐way ANOVA were then conducted to compare differences in these outcome scores by socio‐demographic, health history and lifestyle variables. Due to the rather small sample size (*N* = 50) and the large number of parallel statistical tests conducted, this was strictly an exploratory approach to bivariate subgroup comparisons as it is well established that substantive conclusions cannot be drawn based on statistical significance testing alone. Moreover, post‐intervention data on oral health knowledge, dental anxiety and oral health self‐management behaviours were analysed using Pearson rank correlation coefficients to assess the direction and strength of associations between independent variables following program completion (H4).

A refined multiple linear regression analysis was performed to determine the major contributors for dental anxiety following intervention without an associated risk of model overfitting and/or unpredictable estimates. The model was limited to just five key predictors among the highest priorities: core intervention pathways (post‐intervention oral health knowledge and behaviours) plus key clinical behavioural correlates (frequency of toothbrush replacement; tooth loss; smoking status). The regression model statistical assumptions were tested rigorously. We verified that the linearity and homoscedasticity assumptions were appropriate by visually inspecting the residual plot of standardized rescaled values versus standardized predicted values.

Outlier distorting effects were ruled out by performing descriptive diagnostic screening of influential observations for all 50 participants (using Cook's distance); with a maximum value of (0.465), which is lower than the conventional cut‐off (> 1.0). The independence of residuals was confirmed with a Durbin‐Watson statistic of 1.988, showing an absence of autocorrelation. This achieved a stable 10:1 participant‐to‐predictor ratio. Collinearity diagnostics, including Tolerance and Variance Inflation Factors (VIF), were calculated to verify estimate stability with the absence of multi‐collinearity. Other secondary sociodemographic and historical medical factors were excluded from the multivariate model and confined to exploratory bivariate testing. *p* values were considered statistically significant if they had two tails smaller than 0.05.

## 3 Result

Table 3 shows that more than half of the subjects (64%) were between 60 and < 70 years old. Females were the largest group (52%). 66% of subjects were married, and over one‐quarter were illiterate (28%). Less than half of the subjects (40%) were homemakers, and 52% reported insufficient income. Moreover, 54% of subjects consider pensions as the primary source of income. More than half of the subjects (68%) live in rural areas with their spouse. Finally, no statistically significant difference was found between oral health knowledge and the causes of not visiting the dentist was found between subjects' demographic characteristics and dental anxiety scores. However, statistically significant differences were observed between oral health knowledge scores and residence (*t* = 7.593, *p* = 0.008) and source of income (*F* = 5.227, *p* = 0.009). Similarly, statistically significant differences were found between oral health behaviours scores and level of education (*F* = 3.467, *p* = 0.010) and occupation before retirement (*F* = 3.634, *p* = 0.020).

**TABLE 3:** Comparison of dental anxiety, oral health knowledge and oral healthcare behaviour scores according to socio‐demographic characteristics of the study participants (*n* = 50).

Variable	*N*	%	Dental anxiety		Oral health knowledge		Oral health behaviours	
			Mean	Significance	Mean	Significance	Mean	Significance
Age								
60–> 70 years	32	64.0	15.5	*F* = 0.938 *p* = 0.399	6.79	*F* = 0.543 *p* = 0.585	12.5	*F* = 1.739 *p* = 0.187
70–80 years	15	30.0	17.1		6.78		12.0	
More than 80 years	3	6.0	16.3		6.48		15.0	
Gender								
Male	24	48.0	16.0	*t* = 0.059 *p* = 0.809	7.17	*t* = 2.575 *p* = 0.115	12.2	*t* = 0.421 *p* = 0.520
Female	26	52.0	16.3		6.54		12.6	
Marital state								
Single	1	2.0	14.0	*F* = 0.414 *p* = 0.743	7.0	*F* = 0.909 *p* = 0.444	16.0	*F* = 0.609 *p* = 0.612
Married	33	66.0	16.4		6.94		12.3	
Divorced	2	4.0	15.7		6.43		12.4	
Widowed	14	28.0	14.0		8.0		12.5	
Level of education								
Illiterate	14	28.0	18.0	*F* = 1.361 *p* = 0.258	5.93	*F* = 2.135 *p* = 0.079	10.7	*F* = 3.467 *p* = 0.010*
Read and write only	11	22.0	15.1		7.45		14.2	
Primary	8	16.0	15.5		6.82		11.8	
Preparatory	3	6.0	12.6		7.0		14.0	
Secondary	11	22.0	16.2		7.38		13.0	
University	3	6.0	16.3		7.33		13.3	
Occupation before retirement								
Employee	18	36.0	16.1	*F* = 1.013 *p* = 0.396	7.39	*F* = 1.906 *p* = 0.142	13.8	*F* = 3.634 *p* = 0.020*
Housewife	20	40.0	13.8		6.60		12.2	
Private work	5	10.0	15.4		7.00		10.5	
Worker	7	14.0	17.0		6.35		11.9	
Income								
Enough	24	48.0	16.4	*t* = 0.231 *p* = 0.633	6.71	*t* = 0.400 *p* = 0.532	12.7	*t* = 0.560 *p* = 0.458
Not enough	26	52.0	15.8		6.96		12.1	
Source of income								
Children	16	32.0	16.8	*F* = 1.554 *p* = 0.222	6.0	*F* = 5.227 *p* = 0.009*	11.5	*F* = 1.621 *p* = 0.209
Current work	5	10.0	16.3		7.15		12.9	
Pension	27	54.0	13.8		7.57		12.8	
Social assistance	2	4.0	16.1		6.84		12.4	
Residence								
Rural	26	52.0	16.8	*t* = 2.036 *p* = 0.160	6.35	*t* = 7.593 *p* = 0.008*	11.8	*t* = 3.530 *p* = 0.066
Urban	24	48.0	15.3		7.38		13.1	
Living condition								
Alone	6	12.0	16.5	*F* = 0.487 *p* = 0.693	7.0	*F* = 1.819 *p* = 0.157	11.7	*F* = 0.233 *p* = 0.873
With children	10	20.0	16.4		6.96		12.6	
With spouse	34	68.0	14.3		7.50		12.5	

*Note: F* = ANOVA test; *t* = independent *t*‐test.

* Statistically significant at *p* ≤ 0.05.

Table 4 shows that the majority of participants (84%) had at least one chronic disease. However, no statistically significant differences were observed in dental anxiety, oral health knowledge or oral health behaviours according to chronic disease status (*F* = 0.706, *p* = 0.499; *F* = 1.379, *p* = 0.262; and *F* = 2.462, *p* = 0.096, respectively). Less than half of them (42%) do not brush their teeth regularly. 54% of subjects ate sweets more than 1 day per week, and approximately three‐quarters drank water < 2 L per day. More than one‐third of subjects never replaced their toothbrush. 62% of subjects didn't visit the dentist during the last year, one‐third of them had no complaint or reason for a visit, while 14% visited the dentist due to a toothache. Moreover, 40% of subjects had an excellent self‐tooth assessment. More than half of the subjects (64%) have partial tooth loss without dentures. However, only 24% of the subjects have dentures, but only 16.67% of them clean their dentures once per day. Finally, a statistically significant difference was found between dental anxiety and tooth loss (*F* = 2.950, *p* = 0.042). A statistically significant difference was found between oral health knowledge and the causes of not visiting the dentist within the last year (*F* = 3.443, *p* = 0.024). In contrast, no statistically significant differences were found between oral health behaviours and health history or lifestyle variables.

**TABLE 4:** Comparison of dental anxiety, oral health knowledge and oral healthcare behaviour scores according to health history and lifestyle characteristics of the study participants (*n* = 50).

Variable	*N*	%	Dental anxiety		Oral health knowledge		Oral health behaviours	
			Mean	Significance	Mean	Significance	Mean	Significance
Chronic disease								
No	8	16.0	15.5	*F* = 0.706 *p* = 0.499	7.17	*F* = 1.379 *p* = 0.262	12.6	*F* = 2.462 *p* = 0.096
Hypertension	26	52.0	16.1		6.83		12.9	
Diabetes	16	32.0	17.4		6.22		10.7	
CVD diseases	4	8.0	16.1		6.84		10.8	
Liver and GIT diseases	8	16.0	15.8		6.23		11.8	
Musculoskeletal diseases	20	40.0	16.2		6.19		12.4	
Endocrine diseases	5	10.0	17.2		6.42		12.6	
Respiratory diseases	9	18.0	16.2		6.60		11.7	
Urinary and renal diseases	6	12.0	15.1		6.89		11.7	
Teeth brushing								
One time per day	17	34.0	16.5	*F* = 0.125 *p* = 0.882	7.06	*F* = 0.915 *p* = 0.408	12.4	*F* = 2.672 *p* = 0.080
Two times per day	12	24.0	15.9		7.08		13.8	
Not brushing their teeth	21	42.0	15.9		6.52		11.7	
Eating sweets								
One day per week	23	46.0	16.2	*t* = 0.026 *p* = 0.873	6.67	*t* = 0.891 *p* = 0.350	12.5	*t* = 0.029 *p* = 0.866
More than 1 day per week	27	54.0	16.1		7.04		12.4	
Drinking water								
< 2 L per day	39	78.0	16.5	*t* = 1.658 *p* = 0.204	6.90	*t* = 0.292 *p* = 0.591	12.2	*t* = 1.072 *p* = 0.306
2 L per day	11	22.0	14.8		6.64		13.1	
Rinse your mouth after a meal								
One time per day	4	8.0	16.7	*F* = 0.654 *p* = 0.524	6.63	*F* = 0.584 *p* = 0.562	12.1	*F* = 1.223 *p* = 0.304
Two times per day	24	48.0	15.6		7.0		13.1	
Three times per day	22	44.0	15.3		7.25		11.5	
Frequency of toothbrush replacement								
One month to < 6 months	5	10.0	15.0	*F* = 1.663 *p* = 0.188	7.0	*F* = 0.543 *p* = 0.655	14.5	*F* = 2.348 *p* = 0.085
Six months to < 1 year	11	22.0	15.2		6.50		11.7	
Annually	13	26.0	16.4		7.0		12.5	
Never	21	42.0	18.2		7.09		12.1	
Visiting the dentist within the last year								
No	31	62.0	15.5	*t* = 0.763 *p* = 0.387	7.16	*t* = 1.588 *p* = 0.214	12.3	*t* = 0.091 *p* = 0.764
Yes	19	38.0	16.5		6.65		12.5	
Causes of not visiting the dentist within the last year								
High cost	11	22.0	15.5	*F* = 0.808 *p* = 0.496	7.16	*F* = 3.443 *p* = 0.024*	12.3	*F* = 0.098 *p* = 0.961
A long time of waiting	1	2.0	15.9		7.11		12.6	
No one was available to come with the patient	1	2.0	16.9		6.27		12.2	
No complaints or cause	18	36.0	19.5		4.5		13.0	
Causes of visiting the dentist within the last year								
Gingivitis or bleeding	3	6.0	16.51	*F* = 0.267 *p* = 0.849	6.65	*F* = 0.992 *p* = 0.405	12.51	*F* = 2.090 *p* = 0.114
Periodic check‐up	1	2.0	15.0		6.0		10.0	
Teeth decay	3	6.0	15.32		8.0		13.61	
Teeth loss	5	10.0	15.84		7.11		13.31	
Teeth ache	7	14.0	16.18		6.84		12.42	
Self‐teeth assessment								
Poor	11	22.0	16.10	*F* = 1.571 *p* = 0.209	7.05	*F* = 0.465 *p* = 0.708	12.75	*F* = 2.295 *p* = 0.090
Fair	10	20.0	18.09		6.82		10.91	
Good	20	40.0	15.50		6.40		12.40	
Very good	9	18.0	14.56		6.89		13.77	
Teeth loss								
No	6	12.0	15.38	*F* = 2.950 *p* = 0.042*	6.84	*F* = 0.433 *p* = 0.731	12.68	*F* = 0.234 *p* = 0.872
Partial denture	7	14.0	15.0		6.80		12.2	
Partial without denture	32	64.0	19.71		6.43		11.85	
Total with denture	5	10.0	17.0		7.33		12.16	
Denture								
No	38	76.0	18.0	*t* = 2.988 *p* = 0.090	6.30	*t* = 1.880 *p* = 0.177	11.80	*t* = 0.790 *p* = 0.379
Yes	12	24.0	15.68		6.98		12.62	
Denture cleaning (no = 12)								
No	2	16.67	16.83	*F* = 0.931 *p* = 0.433	6.0	*F* = 1.226 *p* = 0.311	11.50	*F* = 0.459 *p* = 0.712
One time per day	2	16.67	19.0		6.50		11.75	
One time per day, and rinse after each meal	3	25.0	15.78		7.05		12.70	
One time per week	5	41.66	15.33		6.33		12.33	

*Note: F* = ANOVA test; *t* = independent *t*‐test.

* Statistically significant at *p* ≤ 0.05.

In Table 5, a paired sample *t*‐test identified that there was a statistically significant improvement in subjects' scores regarding dental anxiety, oral health knowledge and oral health behaviours before and after the intervention. Dental anxiety scores decreased significantly from baseline (Mean difference = 17.38, 95% CI [−18.97, −15.79], *t* = −21.95, *p* < 0.001), yielding an exceptionally large effect size (Cohen's *d* = 3.10). Concurrently, a substantial increase was observed for oral health knowledge (Mean difference = 3.96, 95% CI [3.58, 4.34], *t* = 21.15, *p* < 0.001, Cohen's *d* = 2.99) and increase also within oral healthcare behaviours (Mean difference = 2.92, 95% CI [2.09, 3.75], *t* = 7.11, *p* < 0.001, Cohen's *d* = 1.01). This intervention contributes to decreasing anxiety scores among geriatric patients regarding dental care.

**TABLE 5:** Assessment and comparison of dental anxiety, oral health knowledge and behaviours among studied subjects before and after interventions (*n* = 50).

Variable	Pre	Post	Mean difference	95% CI	*T*	*p*	Effect size (Cohen's *d*)
Dental anxiety	33.52 ± 5.94	16.14 ± 3.88	−17.38	[15.79, 18.97]	21.952	< 0.001*	3.104
Oral health knowledge	2.88 ± 1.38	6.84 ± 1.41	3.96	[3.58, 4.34]	21.146	< 0.001*	2.99
Oral healthcare behaviours	9.54 ± 1.83	12.46 ± 2.62	2.92	[2.09, 3.75]	7.106	< 0.001*	1.005

*Note: t* = paired sample *t*‐test.

Abbreviation: CI, confidence interval.

* Statistically significant at *p* ≤ 0.05.

Regarding the correlation matrix between dental anxiety, oral health knowledge and behaviours, which is presented in Table 6 using post‐intervention data, there is a strong positive correlation between oral health knowledge and oral health behaviours (*r* = 0.314, *p* = 0.026). This indicates that when knowledge of oral health care improves, the behaviours of older adults will improve.

**TABLE 6:** Correlation matrix between dental anxiety, oral health knowledge and behaviours.

Variables	Dental anxiety	Oral health knowledge
Oral health knowledge	*R* = −0.082 *p* = 0.572	—
Oral health behaviours	*R* = −0.123 *p* = 0.395	*R* = 0.314 *p* = 0.026*

Abbreviation: *r*, Pearson correlation test.

* Statistically significant at *p* ≤ 0.05.

In the multiple linear regression analysis in Table 7, the overall model was statistically significant (*F* = 2.571, *p* = 0.040), accounting for 22.6% of the variance in dental anxiety scores (*R* = 0.475, *R* ^2^ = 0.226, Durbin‐Watson = 1.988). The adjusted *R* ^2^ was 0.138, indicating that after penalizing for the number of predictors, the model accounted for 13.8% of the variance in the outcome. Among the evaluated predictors, frequency of toothbrush replacement was a significant independent predictor of dental anxiety scores (*β* = 0.291, *t* = 2.133, *p* = 0.039), with less frequent toothbrush replacement associated with higher dental anxiety scores. Similarly, tooth loss was a significant independent predictor of dental anxiety scores (*β* = 0.320, *t* = 2.342, *p* = 0.024), with a greater number of missing teeth associated with higher dental anxiety scores. Collinearity diagnostics showed low levels of multicollinearity, with VIF values ranging from 1.054 to 1.187, indicating that multicollinearity was unlikely to substantially affect the regression estimates.

**TABLE 7:** Best‐fitting multiple linear regression model for dental anxiety.

Predictors	Unstandardized coefficients		Standardized coefficients	*T*	Sig.	95.0% confidence interval for *B*		Collinearity statistics	
	*B*	SE	Beta			Lower bound	Upper bound	Tolerance	VIF
(Constant)	9.610	4.361		2.204	0.033	0.821	18.399		
Frequency of toothbrush replacement	1.114	0.522	0.291	2.133	0.039	0.061	2.167	0.943	1.060
Teeth loss	1.125	0.480	0.320	2.342	0.024	0.157	2.094	0.943	1.060
Smoking	2.239	1.308	0.233	1.712	0.094	−0.396—	4.875	0.949	1.054
Oral health knowledge	−0.278—	0.393	−0.100—	−0.707—	0.483	−1.069—	0.514	0.870	1.149
Oral health behaviours	−0.031—	0.214	−0.021—	−0.146—	0.884	−0.463—	0.400	0.842	1.187

*Note: R* = 0.475, *R* ^2^ = 0.226, Adjusted *R* ^2^ = 138, *F* = 2.571, Significance (*p*) = 0.040 Durbin‐Watson = 1.988.

Abbreviations: β, standardized regression coefficient; *B*, unstandardized regression coefficient; *p*, probability value; SE, standard error; *t*, *t*‐statistic.

## 4 Discussion

Maintaining oral health is essential to the health and well‐being of older adults and is an important determinant of quality of life. Older adults commonly experience tooth loss, dental caries, periodontal disease, dry mouth and oral cancer. However, oral health is often overlooked because of the multiple comorbidities that commonly affect this population. Dental anxiety and financial constraints may further limit access to dental care (Janto et al. 2022). Therefore, this study aimed to assess within‐group changes in dental anxiety, oral health knowledge and oral health behaviours following an oral health literacy intervention among older adults with dental diseases. The findings also provide insight into demographic, socioeconomic and clinical factors associated with oral health knowledge and behaviours in this population.

The present study showed that dental anxiety did not differ significantly according to the demographic characteristics examined, whereas oral health knowledge differed significantly according to residence and source of income. These findings indicate that oral health knowledge varied across some socioeconomic and residential categories within the study sample. One possible explanation is that differences in socioeconomic resources, access to health information or proximity to healthcare services may contribute to these variations. However, these potential explanations were not directly examined in the present study. Moreover, the subgroup findings should be interpreted cautiously as exploratory observations rather than conclusive evidence, particularly given the limited number of participants in some sociodemographic categories.

Recent evidence indicates that oral health literacy among older adults is influenced by socioeconomic circumstances and is associated with several oral health outcomes and preventive behaviours (Bøtchiær et al. 2024). Therefore, oral health education should be accessible to older adults regardless of their socioeconomic or residential circumstances.

Significant differences in oral health behaviour were also observed according to educational level and occupation before retirement. One possible explanation is that educational attainment may influence the ability to understand preventive recommendations and apply health information in daily life. Similarly, previous occupation may reflect differences in socioeconomic circumstances and previous exposure to health information. However, these potential explanations were not directly assessed in the present study, and the observed differences do not establish the mechanisms underlying variations in oral health behaviour. A recent scoping review similarly reported associations between oral health literacy and several oral health behaviours and outcomes among older adults, particularly tooth‐brushing practices, although the strength and consistency of these associations varied across populations (Yu et al. 2024).

The present study found a decrease in dental anxiety scores and increases in oral health knowledge and oral health behaviour scores following the oral health literacy intervention. Several features of the intervention may have contributed to these observed changes, although their individual contributions were not assessed separately. The baseline assessment of oral health knowledge using the Oral Health Knowledge Scale (OHK) helped identify participants' existing knowledge and informed the educational content. The intervention also included detailed explanations, individualized education, practical guidance and an oral‐care brochure. These components may have helped participants better understand their oral health needs and preventive practices. In addition, addressing participants' concerns about dental care may have supported their psychological preparation for dental visits. Nevertheless, the present study design does not allow the effects of individual intervention components to be distinguished or the observed changes to be attributed exclusively to the intervention.

The observed changes within‐group are consistent with broader evidence supporting educational approaches to oral health promotion among older adults. Bashirian et al. (2023), in a systematic review of 23 experimental and quasi‐experimental studies, concluded that oral and dental health education interventions generally improved oral health outcomes among older people. Similarly, AlSwayyed et al. (2017) and Shafa Khah and Behzadi (2021) reported that brief educational interventions, including educational videos provided before non‐surgical endodontic procedures and among patients undergoing root canal treatment, were associated with reductions in dental fear and anxiety. El Hajj et al. (2021) also reported that higher educational attainment was associated with lower dental anxiety among Lebanese adults, whereas Ebrahimi and Farokhi (2022) found that health education tailored to patients' needs improved oral health literacy. In contrast, Badran et al. (2023) found no statistically significant association between oral health literacy and dental anxiety in Egypt. Janrak et al. (2023) similarly found no significant association between oral health literacy and health information‐seeking behaviour among older adults in Thailand. These differences may reflect variations in population characteristics, measurement instruments, intervention approaches and healthcare contexts.

The descriptive findings indicate that the participants experienced several oral health challenges beyond knowledge deficits. A considerable proportion did not brush their teeth regularly, frequently consumed sweets, drank < 2 L of water per day, did not replace their toothbrush regularly, and had not visited a dentist during the previous year. Partial tooth loss without dentures was also common. These findings describe the oral health practices and conditions reported by participants but do not establish the reasons underlying these patterns. Although most individual lifestyle characteristics were not significantly associated with oral health behaviour in the bivariate analyses, this lack of statistical significance should not be interpreted as evidence that these practices are clinically unimportant. The relatively small sample and the distribution of participants across multiple behavioural categories may have limited the ability to detect associations; however, the extent of any limitation in statistical power cannot be established from these findings alone.

A significant positive correlation was found between oral health knowledge and oral health behaviours (*r* = 0.314, *p* = 0.026). This indicates that greater oral health knowledge was associated with better oral health behaviours, although the magnitude of the association was modest. Oral health literacy extends beyond factual knowledge and involves the ability to obtain, process, understand, and apply health information and services to make appropriate oral health decisions. Therefore, increasing oral health knowledge may facilitate healthier behaviours, but knowledge alone is unlikely to account for all behavioural variation. Economic resources, physical ability, established habits, access to dental services, family support, perceived need for treatment, and dental anxiety may also influence oral health practices. Consequently, oral health education should be considered an important component of behavioural improvement rather than the sole determinant of behaviour. This interpretation is consistent with Yu et al. (2024), who reported associations between oral health literacy and several oral health behaviours and outcomes among older adults while also noting considerable variability across studies. Similarly, Khamrin et al. (2021) found a positive association between oral health literacy and oral healthcare behaviours among older people. Studies from China and Japan also reported significant relationships between oral health literacy and behaviours such as tooth‐brushing duration, fluoride toothpaste use and regular dental checkups (An, Li, et al. 2022; Ueno et al. 2013). Conversely, Sukhabogi et al. (2020) and Firmino et al. (2018) reported no significant association between oral health literacy and some oral health behaviours, particularly dental visit frequency. Thus, the current finding adds to evidence suggesting that oral health knowledge and literacy may support healthier behaviours, while also highlighting the multifactorial nature of behavioural change.

Another important finding was the significant association between tooth loss and dental anxiety. Participants with different patterns of tooth loss demonstrated significantly different dental anxiety scores. Several explanations are possible. Previous painful or distressing dental experiences associated with tooth loss may contribute to subsequent dental anxiety. Conversely, dental anxiety may be associated with postponement or avoidance of dental treatment, potentially allowing oral health problems to progress. However, the cross‐sectional associations between tooth loss and dental anxiety in the present analysis do not establish the direction of this relationship or confirm a bidirectional mechanism. Previous research among older adults has similarly reported associations between dental anxiety, poorer oral status, tooth loss and treatment needs (Elter et al. 1997). Pohjola et al. (2008) also reported associations between dental fear and indicators of dental condition, including missing teeth, with relationships varying according to age. Accordingly, tooth loss may be a clinically relevant marker for identifying older adults who could benefit from assessment of dental anxiety, although this implication requires consideration in the context of the wider evidence.

The multiple regression analysis identified tooth loss as the strongest statistically significant positive predictor of dental anxiety in the fitted model (*β* = 0.320, *p* = 0.024). This association indicates that tooth loss was related to higher dental anxiety scores after accounting for the other variables included in the model. One possible explanation is that previous difficult or painful dental experiences may contribute to anxiety among individuals with tooth loss. However, the present study did not directly assess the participants' histories of painful extraction procedures or establish whether such experiences accounted for the observed association. Therefore, this explanation should be considered a hypothesis rather than a finding demonstrated by the study.

Infrequent toothbrush replacement was also a statistically significant positive predictor of dental anxiety (*β* = 0.291, *p* = 0.039). The reasons for this association remain uncertain. Infrequent toothbrush replacement might reflect differences in oral self‐care practices or access to preventive resources; alternatively, it may be unrelated to dental anxiety through the proposed mechanisms. The study did not directly assess self‐efficacy, avoidance of self‐care, feelings of shame or anticipated judgment by dental professionals. Consequently, these explanations cannot be confirmed from the available data.

Post‐intervention oral health knowledge and oral health behaviour did not show statistically significant independent associations with dental anxiety in the fitted regression model. This finding suggests that these variables did not demonstrate independent statistical associations with dental anxiety after adjustment for the other included variables. It does not establish that oral health literacy has a secondary causal role or that knowledge and behaviour are unimportant for managing dental anxiety. Although the intervention was followed by improvements in knowledge and behaviour scores, the correlation between these two variables (*r* = 0.314, *p* = 0.026) represents an association rather than evidence that the intervention caused both changes through a particular mechanism. The regression model had an adjusted *R* ^2^ of 0.138, indicating that it explained a modest proportion of the variance in dental anxiety. Therefore, the identified predictors should be interpreted cautiously, and other unmeasured factors may also be relevant.

Previous studies have reported associations between dental anxiety and poorer oral status, greater numbers of missing teeth and greater treatment needs among older adults (Elter et al. 1997; Pohjola et al. 2008). These findings are compatible with the possibility that dental anxiety and poor oral health may coexist and potentially reinforce one another. For example, anxiety‐related avoidance of dental care could contribute to delayed treatment, while adverse dental experiences may be associated with subsequent anxiety. However, the present study did not establish this proposed cycle or determine the temporal relationship between dental anxiety and deterioration in oral health. Screening for dental anxiety may nevertheless be worth considering among older adults with extensive tooth loss or irregular dental attendance. Although the overall regression model was statistically significant (*F* = 2.571, *p* = 0.040), its adjusted *R* ^2^ of 0.138 indicated modest explanatory capacity. Accordingly, the regression findings should be interpreted as associations within the present sample rather than evidence of causal relationships.

Taken together, the findings highlight the coexistence of socioeconomic, educational, behavioural, clinical and psychological factors relevant to oral health among older adults. Although oral health knowledge and behaviour scores improved following the intervention, the study design does not establish the extent to which the intervention alone accounted for these changes. The observed associations with dental anxiety also indicate that oral health knowledge and behaviour may not fully explain individual differences in anxiety. These findings support consideration of educational interventions alongside individualized assessment of dental anxiety, reinforcement of preventive oral health practices and attention to potential barriers to routine dental care. Further studies using controlled designs and larger samples are needed to confirm the observed changes, investigate the proposed explanations and clarify the relationships between oral health literacy, dental anxiety and oral health outcomes.

## 5 Conclusion

The findings of this study suggest that dental anxiety scores decreased, while oral health knowledge and everyday oral health behaviour scores increased following the oral health literacy intervention among older adults with dental diseases. The positive relationship between oral health knowledge and behaviour also suggests that higher levels of oral health knowledge may be associated with better self‐care among older adults.

At the same time, the results show that oral health in later life is influenced by more than knowledge alone. Financial circumstances, previous oral health problems, lifestyle habits and dental anxiety may all affect how older adults approach dental care.

## 6 Implications for Nursing Practice

Oral health assessment in older adults should extend beyond identifying dental pain or visible oral problems. Dental anxiety, oral hygiene practices, previous dental experiences, tooth loss and understanding of oral health should also be considered. Older adults with extensive tooth loss, irregular dental attendance, financial difficulties, smoking habits or poor oral hygiene practices may require individualized support. For some patients, providing information alone may be insufficient; reassurance, practical demonstrations, repeated education and assistance in overcoming barriers to dental care may be required.

Gerontological nurses are particularly well positioned to identify these needs because of their regular contact with older adults in outpatient clinics, community services, primary healthcare facilities and long‐term care settings. Incorporating brief oral health education and dental anxiety screening into routine geriatric assessment may facilitate earlier identification of oral health problems and support timely referral to dental professionals. Integrating oral health standards and training into nursing curricula may also enhance nurses' competence in assessing, planning, implementing and evaluating oral care. A narrative review highlighted the importance of incorporating oral care into nursing practice in care homes to normalize oral hygiene practices and improve coordination with dental professionals (Mac Giolla Phadraig et al. 2022). Similarly, de Lacerda Vidal et al. (2018) reported that a nurse‐led intervention improved oral hygiene among hospitalized older patients. Strengthening oral health education within nursing education may therefore contribute to improved oral and overall health outcomes among older adults (Raftery et al. 2023).

### 6.1 Strengths and Limitations

One strength of this study is its multidimensional assessment of oral health, incorporating dental anxiety, oral health knowledge and everyday oral health behaviours rather than focusing on knowledge alone. The pre‐ and post‐intervention assessments also enabled the assessment of changes following the educational sessions. In addition, the intervention was adapted to participants' needs and educational levels, which may have facilitated understanding and practical application of the information.

Several limitations should be considered when interpreting the findings. First, the use of a convenience sample recruited from a dental outpatient clinic may have introduced selection bias and may limit the generalizability of the findings to older adults in the wider community. In particular, older adults with severe dental anxiety who tend to avoid or delay dental care may have been less likely to attend the clinic and, therefore, may have been underrepresented in the sample. Conversely, older adults who were able or motivated to seek dental care may have been more likely to participate. This is particularly important because dental anxiety was one of the primary outcomes of the study.

Second, the relatively small sample size may have limited statistical power and the ability to detect associations across multiple demographic and behavioural categories.

Third, the lack of randomization and a control group limits causal inference; therefore, the improvements observed after the intervention cannot be attributed to the educational intervention with complete certainty. Repeated testing, increased familiarity with the healthcare team, or other factors during the study period may have contributed to the observed changes.

Fourth, the study was not prospectively registered in a public clinical trial registry before participant recruitment. Although the study received institutional ethical approval, prospective trial registration was not completed before recruitment commenced. This lack of prospective registration should be considered when interpreting the findings.

Fifth, the study did not directly assess oral health literacy using standardized instruments such as REALD or TOFHLiD. Although these instruments provide broader measures of oral health literacy, their applicability among older adults with different educational levels and visual or cognitive limitations, particularly in Arabic‐speaking contexts, may require further adaptation and validation. Consequently, oral health knowledge and behaviour questionnaires were used as indirect indicators of selected dimensions related to oral health literacy rather than as a direct measure of overall oral health literacy.

Sixth, although forward‐ and back‐translation, expert review and face validity assessment were undertaken to enhance the linguistic equivalence, clarity and appropriateness of the instruments, comprehensive psychometric evidence for the Arabic versions of the OHK, OHB and S‐DAI may be limited specifically among older Arabic‐speaking adults with dental diseases. Therefore, the findings should be interpreted with consideration of this measurement‐related limitation, and further psychometric evaluation of these instruments in this specific population is warranted.

Finally, many outcomes were self‐reported, which may have introduced recall or social desirability bias.

Future studies should therefore include larger and more diverse samples recruited from multiple healthcare and community settings and should use randomized controlled designs where feasible. Prospective registration should also be completed before participant recruitment in future intervention studies. Longer follow‐up periods would help determine whether changes in knowledge, dental anxiety and oral health behaviours are sustained over time. Future research should additionally incorporate validated oral health literacy instruments suitable for Arabic‐speaking older adults together with objective clinical indicators of oral health.

## Author Contributions

**Abeer Ramadan Awade Gamaa:** methodology. **Mohamed Hussein Ramadan Atta:** investigation. **Mohamed Gamal Elsehrawy:** formal analysis. **Nesreen AbdelMonaem AbdelSataar AbouZeid:** supervision. **Naglaa Alsaied Mostafa Alfayomy:** writing – original draft and final draft.

## Funding

This research was funded by Princess Nourah Bint Abdulrahman University Researchers Supporting Project number (PNURSP2026R839), Riyadh, Saudi Arabia.

## Disclosure

Generative AI disclosure: The authors declare that no generative artificial intelligence tools were used to generate or analyse the research data, conduct the statistical analysis or produce the scientific findings reported in this manuscript.

## Ethics Statement

The study adhered to the ethical guidelines outlined in the latest version of the Declaration of Helsinki, updated in Fortaleza in 2013 by the World Medical Association (WMA).

## Consent

The authors have nothing to report.

## Conflicts of Interest

The authors declare no conflicts of interest.

## Data Availability

The data that support the findings of this study are available from the corresponding author upon reasonable request, subject to applicable ethical and institutional restrictions.
